# Alterations in resting‐state functional connectivity in pediatric patients with tuberous sclerosis complex

**DOI:** 10.1002/epi4.12523

**Published:** 2021-08-03

**Authors:** Oleg V. Lobanov, Joshua S. Shimony, Jeanette Kenley, Sydney Kaplan, Dimitrios Alexopoulos, Jarod L. Roland, Matthew D. Smyth, Christopher D. Smyser

**Affiliations:** ^1^ Department of Neurology Washington University St. Louis MO USA; ^2^ Department of Radiology Washington University St. Louis MO USA; ^3^ Department of Neurological Surgery University of California San Francisco San Francisco CA USA; ^4^ Department of Neurological Surgery Washington University St. Louis MO USA; ^5^ Department of Pediatrics Washington University St. Louis MO USA

**Keywords:** epilepsy, epilepsy surgery, functional connectivity, functional MRI, tuberous sclerosis complex

## Abstract

**Objective:**

To investigate resting‐state functional connectivity (FC) in pediatric patients with tuberous sclerosis complex and intractable epilepsy requiring surgery.

**Methods:**

Resting‐state functional MRI was utilized to investigate functional connectivity in 13 pediatric patients with tuberous sclerosis complex (TSC) and intractable epilepsy requiring surgery.

**Results:**

The majority of patients demonstrated a resting‐state network architecture similar to those reported in healthy individuals. However, preoperative differences were evident between patients with high versus low tuber burden, as well as those with good versus poor neurodevelopmental outcomes, most notably in the cingulo‐opercular and visual resting‐state networks. One patient with high tuber burden and poor preoperative development and seizure control had nearly normal development and seizure resolution after surgery. This was accompanied by significant improvement in resting‐state network architecture just one day postoperatively.

**Significance:**

Although many patients with tuberous sclerosis complex and medically refractory epilepsy demonstrate functional connectivity patterns similar to healthy children, relationships within and between RSNs demonstrate clear differences in patients with higher tuber burden and worse outcomes. Improvements in resting‐state network organization postoperatively may be related to epilepsy surgery outcomes, providing candidate biomarkers for clinical management in this high‐risk population.

**Key points**

- Severity of impairments in tuberous sclerosis complex (TSC) remain challenging to predict using current diagnostic modalities
- Functional connectivity (FC) was investigated in 13 pediatric patients with TSC and intractable epilepsy requiring surgery
- Resting‐state networks (RSN) demonstrate differences in patients with higher tuber burden and worse neurodevelopmental outcomes
- Early postoperative improvement in RSN organization may be related to epilepsy surgery outcomes
- Improvements in RSN architecture may provide candidate biomarkers for clinical management in this high‐risk population

## 1 INTRODUCTION

Tuberous sclerosis complex (TSC) is an autosomal dominant disorder affecting multiple organ systems, including the brain.^1^ It results from mutations in the TSC1 and TSC2 genes in 9q34 and 16p13, respectively.^2^, ^3^ Its neuroimaging and neuropathological manifestations include cortical tubers (characterized by loss of normal cortical structure and presence of dysmorphic neurons and giant cells), subependymal nodules, and subependymal giant cell astrocytomas.^4^ Neurological manifestations of TSC include seizures, intellectual disability, and neurobehavioral abnormalities.^5^ Of those with epilepsy, up to 62.5% are refractory and subsequently are potential candidates for epilepsy surgery.^6^

Neurodevelopmental outcomes in TSC patients are difficult to predict. Clinical variables related to developmental delays and intellectual disability common in TSC include tuber number and location,^7^, ^8^ tuber‐to‐brain volume proportion,^7^ age at seizure onset,^8^ presence and duration of infantile spasms,^9^ and genetic factors, with TSC2 mutations associated with more severe phenotypes.^10^, ^11^, ^12^ However, the limitations in accurately predicting phenotypes using clinical data are increasingly recognized.^7^, ^13^, ^14^ For example, tuber burden estimation may be affected by scanning sensitivity, lesion identification protocols, and imaging modality.^7^ Further, tubers do not enhance with contrast and can be isointense to adjacent normal tissue. In addition, pathological changes may extend outside MR‐visible “TSC lesions” and involve normal appearing white matter.^15^, ^16^, ^17^

Newer imaging techniques assessing structural and functional connectivity (FC) may play an important role in overcoming these limitations. Studies utilizing diffusion tensor imaging (DTI) to assess white matter tracts demonstrated decreased regional^16^, ^18^, ^19^ and global structural connectivity in TSC patients.^20^ Further, TSC patients with developmental delay had lower structural connectivity indices compared to those without.^20^ In contrast, resting‐state functional magnetic resonance imaging (rs‐fMRI) assesses FC through measurement of infraslow (<0.1 Hz), temporally correlated intrinsic activity to characterize functionally related networks.^21^, ^22^ Assessing brain activity using rs‐fMRI does not require stimuli or participation in tasks and may be performed under sedation.^23^, ^24^

In the only study examining FC in infants with TSC to date, Ahtam et al^23^ identified at least one of the auditory, motor, or visual resting‐state networks (RSNs) in 76.5% of children with TSC. Building upon this work, rs‐fMRI was utilized to investigate FC in pediatric patients with TSC and intractable epilepsy requiring surgery. rs‐fMRI data were acquired before and after epilepsy surgery in 13 patients with TSC. Subjects were categorized based upon tuber burden and developmental outcome approximately 1 year after surgery to investigate FC differences between children grouped by disease severity and cognitive performance. We also investigated whether changes in individual subject's pre‐ and the postoperative rs‐fMRI FC data were related to neurodevelopmental outcomes.

## 2 METHODS

### 2.1 Subjects

Thirteen children (age 1.1‐17 years, mean 5.9) with TSC and medically refractory epilepsy requiring surgery were included. rs‐fMRI data were collected as part of pre‐ and postoperative MRI scans routinely performed before (mean 14 days, range 1‐78) and after (mean 3.5 days, range 1‐13) epilepsy surgery at our institution. Two subjects underwent corpus callosotomy, and another 11 underwent tuberectomy. All aspects of the study were approved by the Human Research and Protection Office Institutional Research Board. Consent was obtained from the parent/legal guardian.

All surgeries were performed by a single pediatric neurosurgeon (MDS). Surgical candidacy was determined by clinical criteria alone. Eleven subjects were sedated for MRI scans with propofol, while two did not receive sedation based on the ability to tolerate nonsedated brain MRI.

### 2.2 Neuroimaging protocol and processing

All imaging was performed using a 3T Siemens Trio scanner and 12‐channel head coil. Structural imaging included T1‐weighted (T1w; repetition time [TR] = 2000 ms, echo time [TE] = 2.5 ms, voxel size 1.0 × 1.0 × 1.0 mm^3^) and T2‐weighted (T2w; TR = 9000 ms, TE = 115 ms, voxel size 1.0 × 1.0 × 2.5 mm^3^) sequences. For clinical reasons, preoperative T1w was acquired with IV contrast at the end of the session. Postoperative T1w was collected without contrast. The remaining sequences were identical across pre‐ and postoperative sessions. rs‐fMRI data were acquired using an echoplanar imaging (EPI) sequence sensitive to blood oxygen level‐dependent contrast (TR = 2070 ms, TE = 25 ms, voxel size 4.0 × 4.0 × 4.0 mm^3^). Two 200 frame runs were acquired in each subject, providing ~14 minutes of data. A diffusion sequence (TR = 8400 ms, TE = 98 ms, voxel size 2.2 × 2.2 × 3 mm^3^) was used to calculate apparent diffusion coefficient (ADC) values for tuber identification and volume calculation.

Preprocessing of the rs‐fMRI data was performed utilizing the 4dfp suite of tools^25^ (https://readthedocs.org/projects/4dfp/). This included correction for asynchronous slice acquisition, normalizing slice intensity, and correction of interframe head motion. Atlas registration was computed via T2w atlas‐representative template using published methodology.^26^ Briefly, preoperative T2w images were registered to a T2w atlas‐representative template.^27^ Both pre‐ and postoperative EPI images were then registered to the preoperative T2w with manual verification of results. The conventional T1w image to T1w atlas template registration was avoided because the preoperative T1w image included contrast, and the postoperative T1w could have anatomical deformation. Following transformation to atlas space, rs‐fMRI data were resampled to 3.0 × 3.0 × 3.0 mm^3^ before time‐series correlation analysis. Frame censoring was performed; motion‐corrupted volumes exceeding a framewise displacement of 0.2 mm were removed.^28^ The rs‐fMRI time series was demeaned and detrended, and nuisance regression was performed including the following: (1) 24‐head motion parameters, (2) white matter and CSF time series, and (3) whole‐brain regressor.^29^ Data were interpolated between epochs removed due to motion, temporally filtered (0.009/0.08 Hz), and spatially smoothed with a 6‐mm FWHM Gaussian kernel. Matlab 2015b was used for subsequent analyses.

### 2.3 Tuber burden and Developmental delay classification

A semiautomated tuber classification algorithm calculated tuber‐to‐brain volume ratio using ADC data, followed by manual verification of tuber burden and classification (OL). Tubers were quantified by applying a threshold (mean + 2 × std) to the ADC image after CSF removal and brain extraction. Total tuber volume was calculated as a voxel sum, with the ratio of total tuber to whole brain volume used to classify subjects as high versus low tuber burden.

Development was classified as no/mild delay versus moderate/severe delay at the time of each patient's last preoperative clinic visit and compared to development at the time of each patient's last postoperative clinic visit (mean 55 months, range 6‐117). Moderate/severe delay was determined based upon developmental delay >33% in a domain as documented at the clinic visit.^30^ All subjects classified as severely delayed met these criteria in at least two domains, satisfying clinical criteria for global developmental delay.^31^

### 2.4 Voxel‐mirrored homotopic connectivity

Voxel‐mirrored homotopic connectivity (VMHC) was computed on the preoperative data as the Fisher Z‐transformed Pearson correlation between the time series of every pair of symmetric interhemispheric voxels.^32^, ^33^, ^34^ VHMC measures connectivity between homotopic counterparts and is dependent on the integrity of interhemispheric connections, mainly the corpus callosum.^26^ Global and regional VMHC in sensorimotor, vision, frontal, parietal, occipital, and temporal areas were calculated.^26^

### 2.5 Functional connectivity

Functional connectivity was calculated on the preoperative data as Fisher z‐transformed Pearson correlation coefficients between the averaged rs‐fMRI signal from regions of interest (ROI), assembled into ROI × ROI matrices, and sorted into one of 12 previously defined RSNs.^35^ A well‐described set containing 300 ROIs was used.^36^ For postoperative data, variable numbers of ROIs falling into the surgical bed were removed from the original set. The resultant set, unique for each subject, was used to generate FC matrices using identical ROIs for pre‐ and postsurgical comparisons. From these matrices, composite measures were calculated for each RSN for group comparisons.^37^

Two sample *t* tests compared FC in low versus high tuber burden groups, as well as no/mild versus moderate/severe delay groups. Given the exploratory nature of these analyses, a significance cutoff of *α* = .05 was utilized for all statistical comparisons.

## 3 RESULTS

Of 13 subjects, five had low and eight had high tuber burden. Similarly, there were five subjects with no/mild and eight with moderate/severe developmental delay. Four subjects with low tuber burden had good developmental outcomes. Seven subjects in the high tuber burden group and no subjects in low tuber burden group were treated with vigabatrin prior to surgical intervention.

### 3.1 Decreased FC in high versus low tuber burden

**FIGURE 1:**
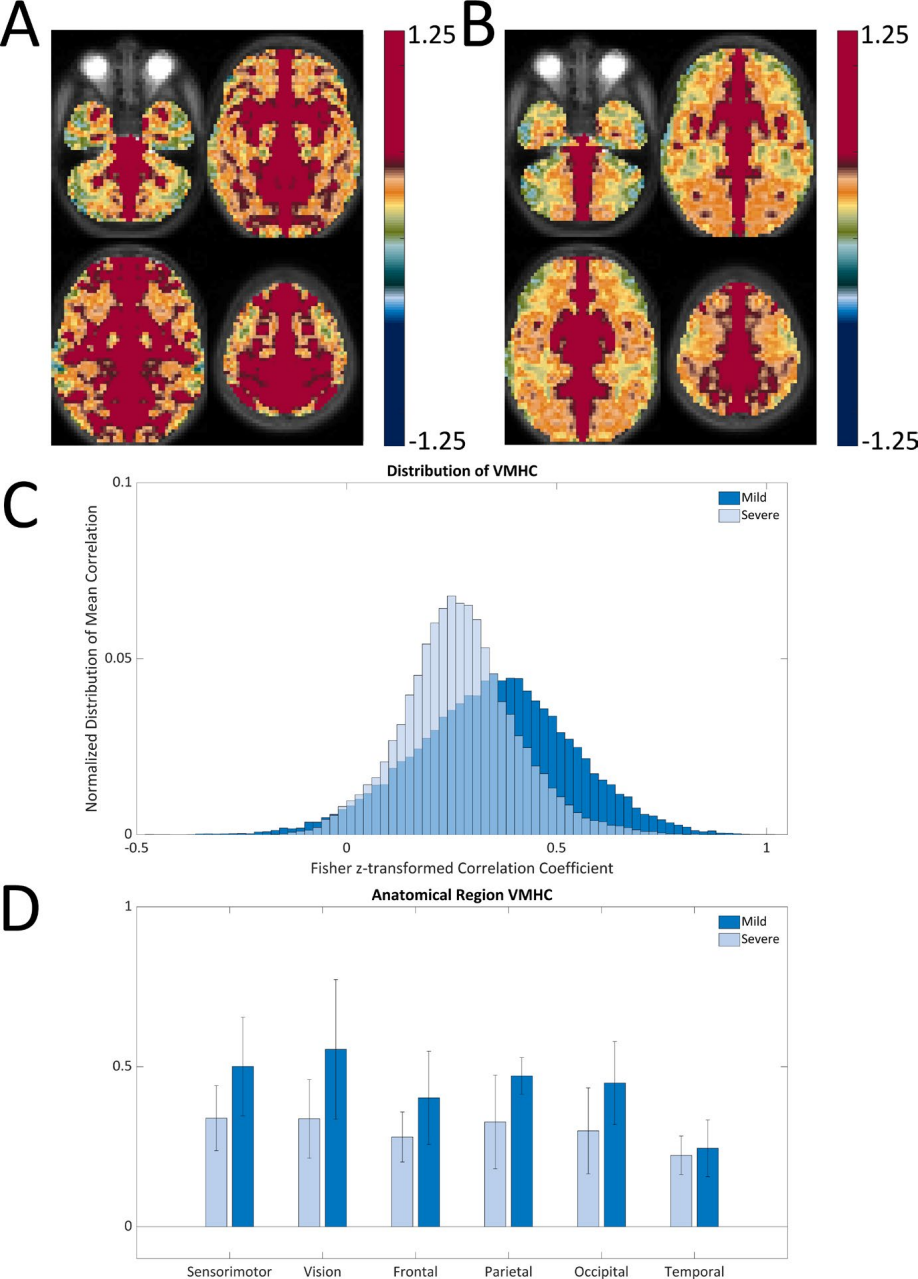
Group mean presurgical voxel‐mirrored homotopic functional connectivity (VMHC) computed as the Pearson correlation (Fisher Z‐transformed) between every pair of symmetric interhemispheric voxel's time series. VMHC is overlaid on a T2‐weighted atlas‐representative image. Higher VMHC values are seen in low (A) compared to high (B) tuber burden groups. This difference is especially notable in frontal and parietal regions. (C) Global distribution is shifted toward zero in high when compared to low tuber burden group. (D) VMHC (mean ±95% confidence interval) organized according to anatomical region. Regional values of VHMC were lower in the high tuber burden group across all regions

Figure 1 demonstrates group mean differences in preoperative VMHC in the low versus high tuber burden groups. Both global (Figure 1C) and regional (Figure 1D) values of VMHC were lower in high tuber burden subjects.

**FIGURE 2:**
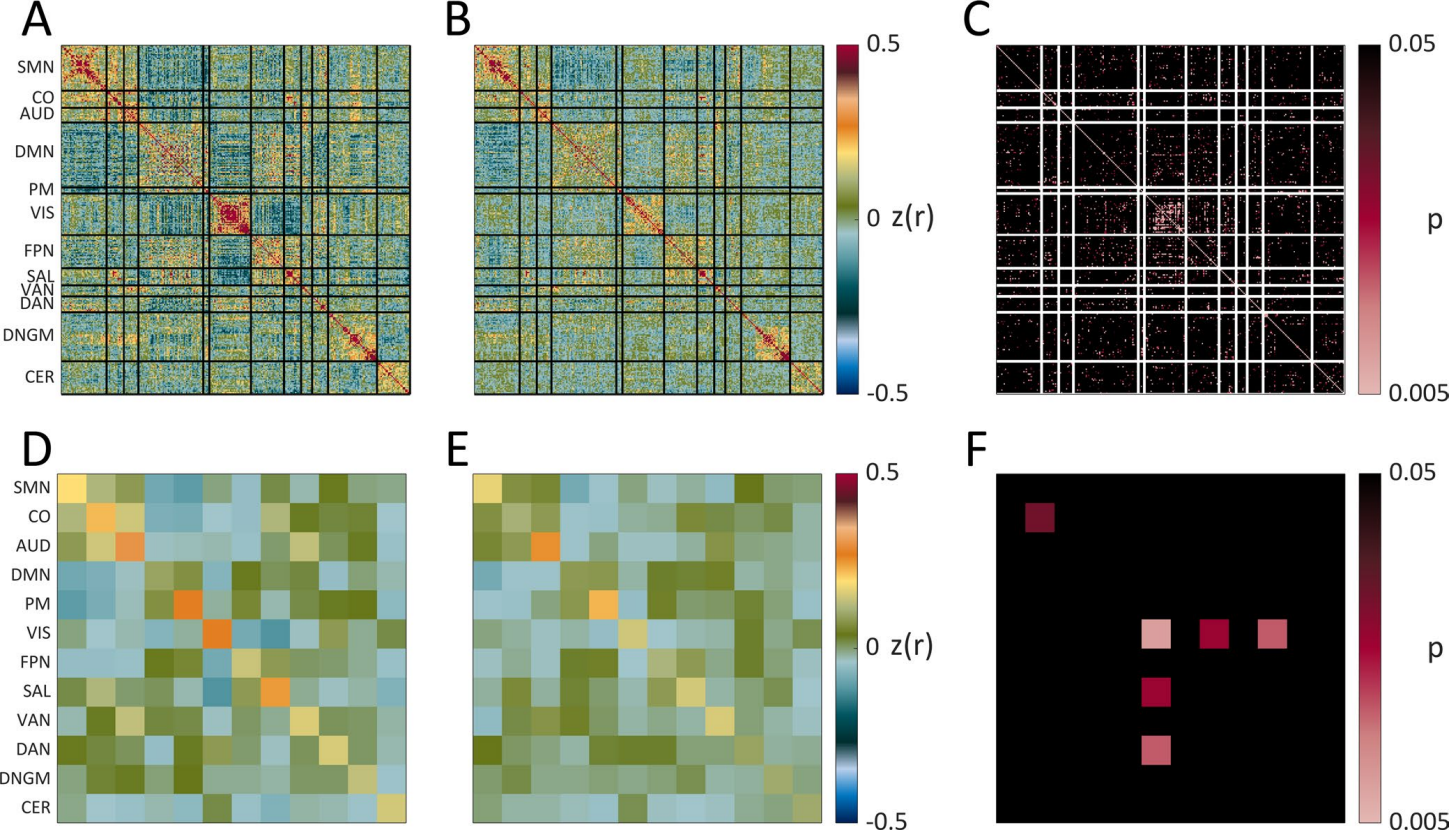
Presurgical FC matrices in low (A) and high (B) tuber burden groups, as well as their direct statistical comparison using two‐sample *t* test (C). The bottom row demonstrates network averages for 12 resting‐state networks in low (D) and high (E) tuber burden groups, as well as their comparison (F). Across both sets of matrices, within‐network connectivity is reflected in on‐diagonal measures, with between‐network connectivity reflected in off‐diagonal measures. AUD, Auditory; CER, Cerebellum; CO, Cingulo‐opercular; DAN, Dorsal attention network; DMN, Default mode network; DNGM, Deep nuclei gray matter; FPN, Frontoparietal network; PM, Parietal memory; SAL, Salience; SMN, Sensorimotor; VAN, Ventral attention network; VIS, Visual

Figure 2 demonstrates group mean preoperative RSN FC in high versus low tuber burden groups. Both groups demonstrate higher magnitude within‐network (ie, on‐diagonal) and lower magnitude between‐network (ie, off‐diagonal) correlations, a typical pattern of RSN organization. Similar results are evident when looking at network averages (2D, 2E). When comparing FC between groups (2C, 2F), FC within the cingulo‐opercular (CO) and visual RSNs and between the visual and dorsal attention RSNs is greater in the low tuber burden group (*P* = .03, .01, and .02, respectively). Conversely, FC between the visual and salience RSNs is lower in the low tuber burden group (*P* = .03).

### 3.2 Decreased FC in moderate/severe versus no/mild delay

Similar to the tuber burden group comparison, decreased preoperative across midline connectivity (VMHC) is seen in frontal and parietal areas in the moderate/severe versus no/mild delay groups. Global and regional VMHC values were similarly lower in the severe delay group. Both groups demonstrate comparable network organization with higher within‐network and lower between‐network correlations. However, FC was higher between the auditory and default mode (DMN) RSNs and lower between the DMN and ventral attention RSNs in the no/mild versus moderate/severe groups (*P* = .04 and .03, respectively).

### 3.3 Pre‐ versus postsurgical FC

**FIGURE 3:**
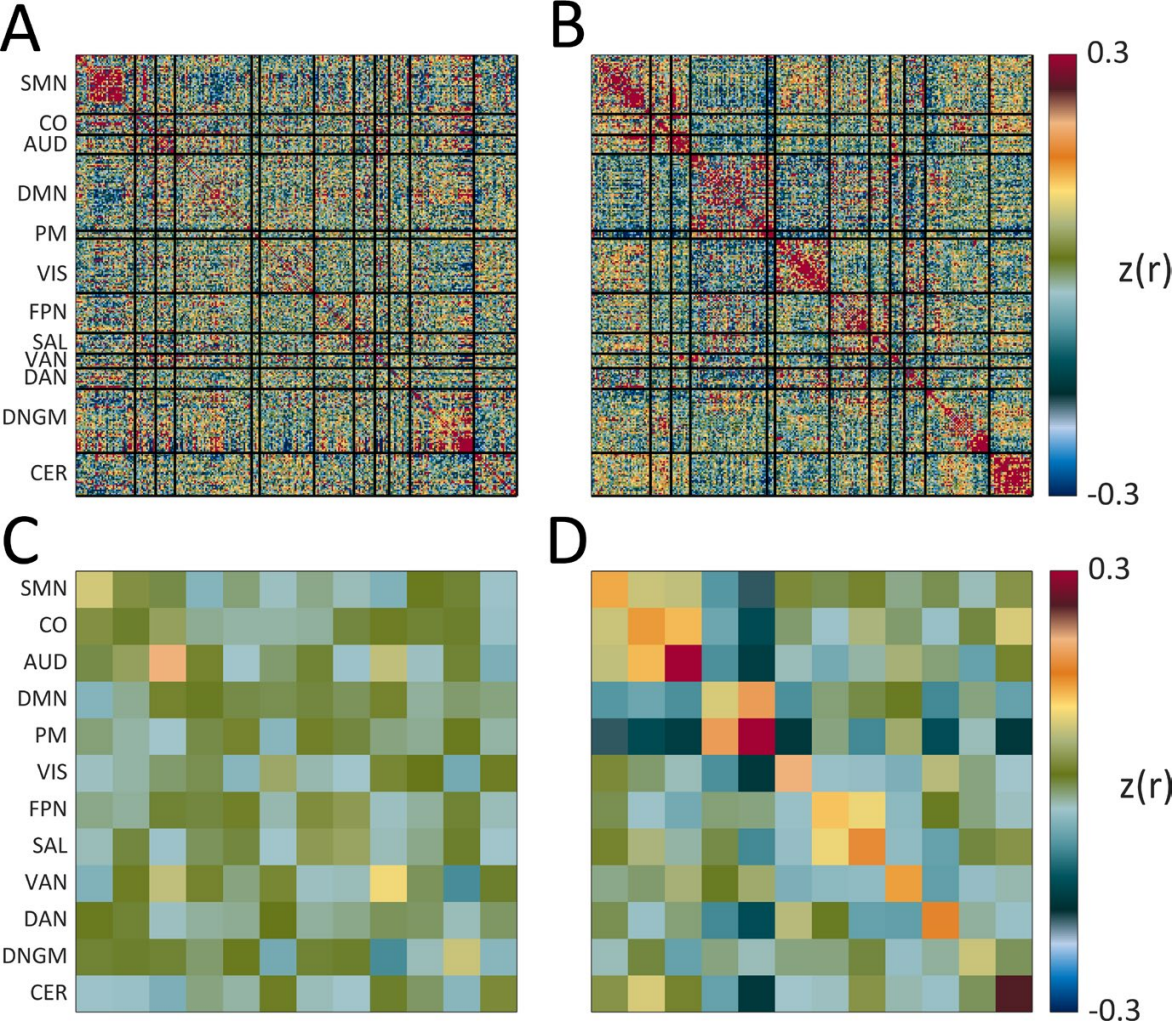
Presurgical (A—all ROIs, C—resting‐state network averages) versus postsurgical (B—all ROIs, D—resting‐state network averages) FC in an individual patient with markedly improved developmental trajectory and seizure resolution after tuberectomy. Improvement in FC was ascertained through assessment of correlation value magnitude (both positive and negative) from pre‐ to postoperative studies. Within network connectivity before and after surgery is: SMN—0.09 (pre) vs 0.14 (post), CO—0.02 (pre) vs 0.15 (post), AUD—0.2 (pre) vs 0.35 (post), DMN—0.02 (pre) vs 0.09 (post), PM—0.03 (pre) vs 0.57 (post), VIS—0.06 (pre) vs 0.21 (post), FPN—0.04 (pre) vs 0.13 (post), SAL—0.06 (pre) vs 0.17 (post), VAN—0.11 (pre) vs 0.15 (post), DAN—0.004 (pre) vs 0.17 (post), DNGM—0.09 (pre) vs 0.09 (post), CER—–0.04 (pre) vs 0.25 (post). AUD, Auditory; CER, Cerebellum; CO, Cingulo‐opercular; DAN, Dorsal attention network; DMN, Default mode network; DNGM, Deep nuclei gray matter; FPN, Frontoparietal network; PM, Parietal memory; SAL, Salience; SMN, Sensorimotor; VAN, Ventral attention network; VIS, Visual

Low‐motion postsurgical rs‐fMRI data were available in six subjects (Table 1). The remaining seven subjects had significant susceptibility artifact from blood products in the postoperative studies preventing successful image registration. When evaluating for differences between pre‐ and postoperative studies, analyses focused upon the magnitude of correlation values (both positive and negative). Overall, three subjects had higher magnitude positive and negative correlations postoperatively, indicative of more typical FC patterns, while achieving seizure freedom and good developmental outcomes. Of those three, two subjects had low tuber burden and mild delay presurgically, whereas one subject had high tuber burden and severe developmental delay preoperatively. Figure 3 demonstrates the pre‐ versus postsurgical FC results in this single subject. To summarize her course, the patient presented at age 5 months with infantile spasms that resolved with vigabatrin. She then developed a new seizure semiology with right‐sided weakness, unresponsiveness, and rapid eye‐blinking occurring up to 17 times/day. These seizures were refractory to medical treatment, and she underwent surgical resection of two tubers at age 1.9 years. Prior to surgery, she was not sitting independently and had no words. Her presurgical FC (18 days prior) is notable for limited within‐network connectivity (Figure 3). Note the improved network architecture just one day after surgery and emergence of typical within‐network connectivity across networks. Two years after surgery, she was seizure‐free, speaking in 6‐8 word sentences, using utensils, and attending regular preschool.

**TABLE 1:** Clinical data for subjects with pre‐ and postsurgical functional connectivity data

Age, years	Sz Type	Infantile spasms	Tuber burden	Surgery	FC change	Engel class	Development pre‐surgery	Development post‐surgery
17	Gen	No	High	CC	NI	IV	Poor	Poor
14.1	Focal	No	Low	Tuberectomy	No data	I	Good	Good
12.1	Focal	No	Low	Tuberectomy	No data	I	Good	Good
8.4	Gen	Yes	High	CC	NI	IV	Poor	Poor
6.6	Focal	No	Low	Tuberectomy	Improved	I	Good	Good
4	Focal	Yes	Low	Tuberectomy	No data	II	Poor	Poor
3.1	Focal	Yes	High	Tuberectomy	No data	II	Poor	Poor
3.1	Focal	No	Low	Tuberectomy	Improved	I	Good	Good
2.6	Focal	Yes	High	Tuberectomy	NI	I	Poor	Poor
1.9	Focal	Yes	High	Tuberectomy	Improved	I	Poor	Good
1.8	Focal	Yes	High	Tuberectomy	No data	IV	Poor	Poor
1.4	Focal	Yes	High	Tuberectomy	No data	IV	Poor	Poor
1.1	Focal	Yes	High	Tuberectomy	No data	IV	Poor	Poor

Abbreviations: CC, corpus callosotomy; Gen, apparent generalized; NI, no improvement; Sz, seizure.

## 4 DISCUSSION

rs‐fMRI data were analyzed in children with TSC and medically refractory epilepsy. The majority of patients demonstrated resting‐state FC features similar to those observed in healthy individuals,^38^, ^39^, ^40^ with anticipated patterns of positive and negative correlations observed within and between RSNs across subjects. Despite these similarities, RSN‐specific differences in preoperative rs‐fMRI data between patients with high versus low tuber burden, as well as good versus poor developmental outcomes, were observed.

The specific RSNs in which differences between groups were identified are notable. The CO network plays an important role in maintaining alertness^41^, ^42^ and more broadly in cognition, being an important task control network.^43^ Further, activity within the CO network has been associated with response speed to auditory and visual targets, relationships modulated by age and disease.^42^, ^44^, ^45^ Thus, differences in CO connectivity may play a critical role in the cognitive impairments common in TSC. Similarly, lower visual network connectivity may be related to the ophthalmological manifestations of TSC. These include optic nerve hamartomas, cortical visual impairments, and visual field deficits.^46^, ^47^ Further investigation is needed to examine the contribution of these risk factors to altered visual network connectivity in TSC.

Voxel‐mirrored homotopic connectivity was higher in patients with lower tuber burden and better developmental outcomes. VMHC relies on structural connectivity across the midline and has been previously shown to depend on integrity of the corpus collosum and significantly decreases after callosotomy.^26^ Importantly, previous studies utilizing DTI have demonstrated aberrant diffusion measures in the splenium and genu of normally appearing corpora collosa in patients with TSC.^16^, ^18^ We hypothesize that FC between homotopic counterparts across the midline is affected by tuber presence, with its decrease related to worse outcomes in TSC.

Interestingly, not all subjects in the severe tuber burden group had poor outcomes. One intriguing possibility to explain this was the effect of successful epilepsy surgery. To test this hypothesis, FC data before and after surgery were analyzed in a subject with high tuber and seizure burden as well as global delay prior to surgery and excellent developmental and seizure control outcome. There was a significant immediate improvement in the FC architecture just one day after surgery, with appearance of FC patterns similar to those in healthy children. One explanation for this rapid change may be that typical FC was suppressed by the ongoing chaotic activity of frequent seizures, and, once the seizure onset area was successfully removed, RSNs resumed typical relationships. This finding is consistent with another study demonstrating excellent developmental outcome after epilepsy surgery in a patient with onset of epileptic encephalopathy around age 4 years.^48^

Importantly, there was another subject in the high tuber burden group with severe delay prior to surgery who achieved seizure freedom postoperatively. However, in this subject, the FC architecture did not improve postoperatively and the developmental trajectory remained poor. The juxtaposition of results between these two cases suggests that FC may be an important mediator of the association between seizure control and developmental outcomes following epilepsy surgery and that relationships between these variables cannot be determined and/or predicted based upon clinical findings alone.

### 4.1 Limitations

This study was performed in a retrospective fashion with data collection over a period of years. This resulted in lack of standardized developmental or seizure burden assessment, differences in relevant clinical variables across groups (eg, vigabatrin use) and variability in rs‐fMRI data collection (eg, sedation use). In addition, high‐quality postsurgical rs‐fMRI data were available in only 6 out of 13 subjects due to the presence of postoperative changes. This small sample size and age distribution prevents making generalized conclusions. Further, the majority of subjects were sedated with propofol. This is a clear but unavoidable limitation of studying individuals at younger ages and/or with severe delays. Fortunately, cortical and subcortical connectivity are preserved under propofol sedation.^24^, ^49^, ^50^ In addition, sedation reduces head motion artifact, increasing quantities of low‐motion data and reducing colored noise due to subject motion. Subjects also underwent two types of surgical procedures, corpus callosotomy and tuberectomy. The decision to proceed with tuberectomy reflects higher certainty regarding seizure onset location, and the procedure subsequently has a higher chance of achieving seizure freedom. Future prospective investigations which build upon this work and include larger, matched clinical samples and standardized methods across domains may address these key considerations.

### 4.2 Clinical relevance and future directions

Many patients with TSC and medically refractory epilepsy demonstrate a similar architecture of cortical and subcortical RSNs to that of healthy pediatric cohorts. However, relationships within and between RSNs demonstrate clear differences in patients with higher tuber burden and worse neurodevelopmental outcomes. Postoperative improvement in RSN organization may be related to neurodevelopmental outcomes following epilepsy surgery, providing a candidate biomarker for more aggressive medication weaning and/or therapy interventions in this high‐risk population. This study provides an important initial step in utilizing advanced neuroimaging techniques to provide greater understanding of the effects of TSC on FC development in patients with refractory epilepsy. Additional larger, prospective studies remain necessary to confirm and extend these findings with the goal of expanding use of these techniques into standard clinical care.

## CONFLICT OF INTEREST

None of the authors has any conflict of interest to disclose. We confirm that we have read the Journal's position on issues involved in ethical publication and affirm that this report is consistent with those guidelines.
